# Novel insight into the underlying dysregulation mechanisms of immune cell-to-cell communication by analyzing multitissue single-cell atlas of two COVID-19 patients

**DOI:** 10.1038/s41419-023-05814-z

**Published:** 2023-04-22

**Authors:** Shijie Qin, Xiaohong Yao, Weiwei Li, Canbiao Wang, Weijun Xu, Zhenhua Gan, Yang Yang, Aifang Zhong, Bin Wang, Zhicheng He, Jian Wu, Qiuyue Wu, Weijun Jiang, Ying Han, Fan Wang, Zhihua Wang, Yuehua Ke, Jun Zhao, Junyin Gao, Liang Qu, Ping Jin, Miao Guan, Xinyi Xia, Xiuwu Bian

**Affiliations:** 1 Institute of Laboratory Medicine, Jinling Hospital, Nanjing University School of Medicine, 210002 Nanjing, Jiangsu China; 2 Laboratory for Comparative Genomics and Bioinformatics, College of Life Science, Nanjing Normal University, 210046 Nanjing, Jiangsu China; 3 Institute of Pathology, Key Laboratory of Tumor Immunopathology, Ministry of Education of China, Southwest Hospital, Third Military Medical University (Army Medical University), 400038 Chongqing, China; 4 Joint Expert Group for COVID-19, Department of Laboratory Medicine & Blood Transfusion, Wuhan Huoshenshan Hospital, 430100 Wuhan, Hubei China; 5 Department of Gastroenterology, Jinling Hospital, Nanjing University School of Medicine, 210002 Nanjing, Jiangsu China; 6 Medical Technical Support Division, the 904th Hospital, 213003 Changzhou, Jiangsu China; 7 Department of Gastroenterology, Daping Hospital, Third Military Medical University (Army Medical University), 400038 Chongqing, China; 8 Department of Laboratory Medicine and Blood Transfusion, the 907th Hospital, 350702 Nanping Fujian, China; 9 Chinese PLA Center for Disease Control and Prevention, 100070 Beijing, China; 10 Pulmonary and Critical Care Medicine, Yancheng No.1 People’s Hospital, 224000 Yancheng, Jiangsu China; 11 Department of Laboratory Medicine, 920 Hospital of the Joint Service Support Force of the Chinese People’s Liberation Army, 650032 Kunming, Yunnan China

**Keywords:** Viral infection, Infection

## Abstract

How does SARS-CoV-2 cause lung microenvironment disturbance and inflammatory storm is still obscure. We here performed the single-cell transcriptome sequencing from lung, blood, and bone marrow of two dead COVID-19 patients and detected the cellular communication among them. Our results demonstrated that SARS-CoV-2 infection increase the frequency of cellular communication between alveolar type I cells (AT1) or alveolar type II cells (AT2) and myeloid cells triggering immune activation and inflammation microenvironment and then induce the disorder of fibroblasts, club, and ciliated cells, which may cause increased pulmonary fibrosis and mucus accumulation. Further study showed that the increase of T cells in the lungs may be mainly recruited by myeloid cells through ligands/receptors (e.g., ANXA1/FPR1, C5AR1/RPS19, and CCL5/CCR1). Interestingly, we also found that certain ligands/receptors (e.g., ANXA1/FPR1, CD74/COPA, CXCLs/CXCRs, ALOX5/ALOX5AP, CCL5/CCR1) are significantly activated and shared among lungs, blood and bone marrow of COVID-19 patients, implying that the dysregulation of ligands/receptors may lead to immune cell’s activation, migration, and the inflammatory storm in different tissues of COVID-19 patients. Collectively, our study revealed a possible mechanism by which the disorder of cell communication caused by SARS-CoV-2 infection results in the lung inflammatory microenvironment and systemic immune responses across tissues in COVID-19 patients.

## Introduction

A large-scale autopsy reports have demonstrated that SARS-CoV-2 infection can cause multiple organ damage, particularly single-cell transcriptome sequencing results have also revealed that SARS-CoV-2 infection can significantly induce the dysregulation of gene expressions in a variety of organs and immune cells besides both lungs and AT2 cells [1–3]. However, how does SARS-CoV-2 infection promote the pulmonary microenvironment change, and the immune response of remote organs, as well as the migration and redistribution of immune cells among distinct tissues is still unclear to date.

Cell–cell communication refers to the transmission of information from one cell to another cell through a medium to result in a corresponding response [4–6]. The cell–cell communication mediated by the ligand/receptor complex plays a vital role in the activation of specific cell signaling [4–6]. Therefore, identifying specific ligand/receptor interactions is very important for understanding cell behavior and response to neighboring and distant cells. A previous study has reported that ANXA1-FPR1 and S100A8/9-TLR4 are main mediators of cellular communication between lung epithelial cells and myeloid cells, and their disorder may be an important cause leading to immune storm and systemic disorders of lung in severe COVID-19 patients [3]. Even so, there are still much unknown about the detailed change of intercellular communication network in the lungs of patients after SARS-CoV-2 infection to date.

In this study, we carried out the single-cell RNA sequencing (scRNA-seq) of lung, blood, and bone marrow from COVID-19 patients, and found that the change of cell communication in tissue or inter-tissue mediated by the receptor/ligand dysregulation might be responsible for facilitating the pulmonary microenvironment disturbance and systemic immune responses of COVID-19 patients. In a word, our present work will be helpful for further understanding the pathogenesis of SARS-CoV-2.

## Results

### Single-cell sequencing and clinical characteristics of two dead COVID-19 patients

**Fig. 1:**
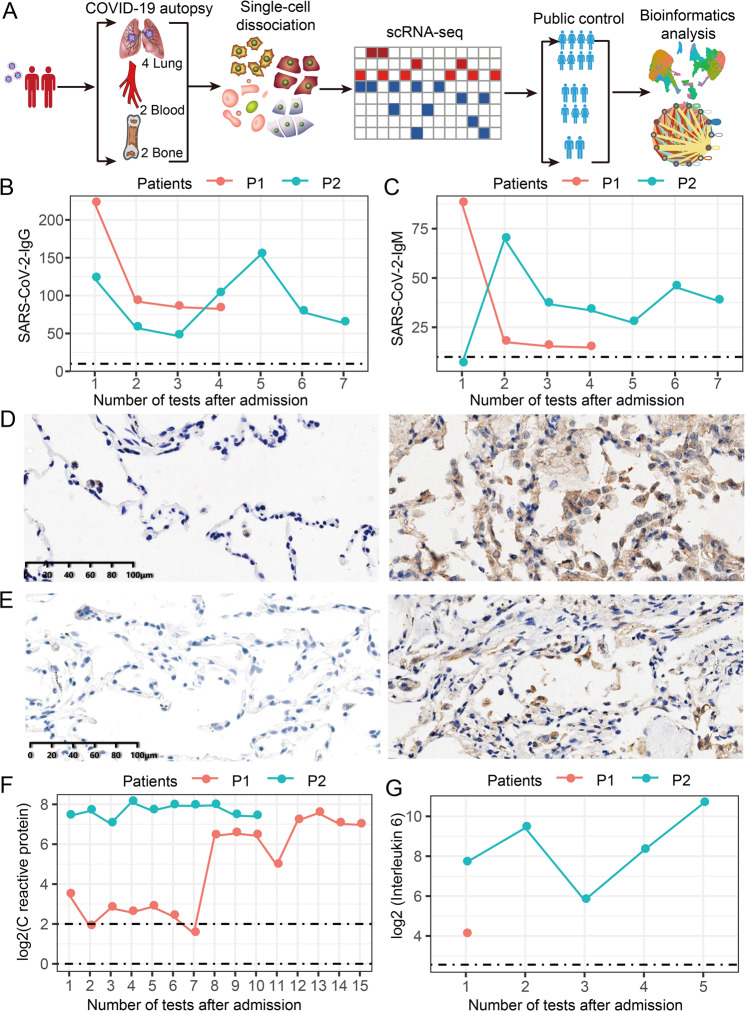
Sampling and single-cell sequencing procedures for two COVID-19 patients. **A** Two cases of COVID-19 patients’ single-cell sequencing and bioinformatics analysis procedures (Table S1). **B**, **C** Detection of SARS-CoV-2-IgG and SARS-CoV-2-IgM in the blood of two COVID-19 patients. The blood of two patients was collected multiple times for testing before death. Below the dotted line represents the normal reference range. **D**, **E** IHC results of SARS-CoV-2 protein in the lungs of age-matched control group and COVID-19 patients. Two controls (C1, C2) on the left side and two dead patients (P1, P2) on the right side (Table S2). **F**, **G** Tests of C-reactive protein and interleukin-6 in the blood of two COVID-19 patients. Below the dotted line or between the dotted line represents the normal reference range.

We herein used the scRNA-seq to characterize these changes in cellular and molecular levels of lungs (left lung and right lung), blood, and bone marrow from the two died COVID-19 patients (Fig. 1A). After quality control and integration of sequenced COVID-19 autopsies and public health samples [7–9], we obtained a total of 110,741 single cell including 21,773 bone marrow cells (17,396 COVID-19 and 4377 healthy), 41,571 blood cells (21,244 COVID-19 and 20,327 healthy), and 47,397 lung cells (4241 COVID-19 and 43,156 healthy) (Fig. 1A). Remarkably, these test results from multiple blood SARS-CoV-2-IgG and SARS-CoV-2-IgM showed that the two COVID-19 patients were significant positive (Fig. 1B, C), indicating that they had been infected by SARS-CoV-2 before they died. In addition, SARS-CoV-2 virus protein was detected in the lungs of the two dead patients by the immunohistochemical (IHC) staining (Fig. 1D, E). Especially, the two COVID-19 patients before death had severe inflammation accompanied by the significant increases of C-reactive protein and interleukin 6 (Fig. 1F, G).

### Lung single-cell atlas and cell communication of dead COVID-19 patients

**Fig. 2:**
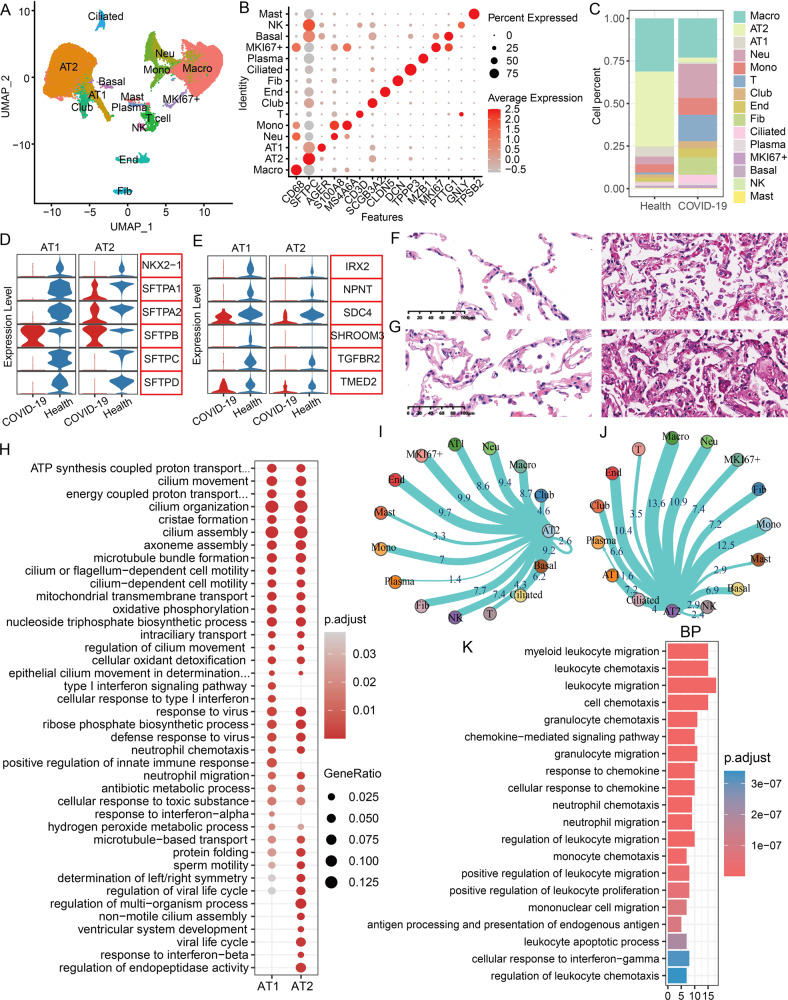
Single-cell transcriptome analysis of the lung organs. **A** The UMAP cluster map shows the cell type clustering results of the lungs of healthy and COVID-19 patients. **B** The expression levels of classic marker genes corresponding to different cell types. **C** The proportion of different cell types in the healthy group and COVID-19 patients. **D** The expression levels of genes that regulate and maintain alveolar structure in the healthy group and COVID-19 patients. **E** The expression levels of genes that maintain epithelial cell homeostasis in the healthy group and COVID-19 patients. **F**, **G** HE staining of the lungs of age-matched control group and COVID-19 patients. On the left are two controls (C1, C2) and on the right are two dead patients (P1, P2). **H** Functional enrichment analysis of the biological processes involved in the differentially expressed genes in AT1/AT2 cells. **I**, **J** The frequency of cell communication between AT2 cells and other cell types in the healthy group and the COVID-19 group. **K** The biological process enrichment of significantly altered ligands and receptors in AT1/AT2 cells.

We herein identified a variety of lung epithelial cells, including AT1 cells (AGER+), AT2 cells (SFTPC+), club cells (SCGB3A2+), ciliated cells (TPPP3+), and basal cells (PTTG1+) (Fig. 2A, B), and found that immune cells were mainly comprised of neutrophils (Neu) (S100A8+), monocyte (Mono) (MS4A6+), T cells (CD3D+), natural killer cells (NK) (GNLY+), plasma cells (MZB1+), mast cells (TPSB2+) and macrophages (Macro) (CD68+) (Fig. 2A, B). Besides, endothelial cells (End) (CLDN5+), fibroblasts (Fib) (DCN+), and MKI67+ cells were also identified (Fig. 2A, B). After infection, these immune cells such as Neu, T, NK, and Mono cells were obviously increased in the lungs (Fig. 2C), but lung macrophages had a slight decrease (Fig. 2C), which was considered to be the excessive consumption of alveolar macrophages caused by infection [10, 11], because alveolar macrophages can be rapidly activated and aggregated early to fight infection [12]. Of note, the ratio of both Fib and club cells were significantly increased (Fig. 2C), but AT1 and AT2 cells were drastically decreased (Fig. 2C). Interestingly, many transcription factors that maintain alveolar structure and integrity, such as NKX2-1, SFTP families, IRX2, NPNT, SDC4, SHROOM3, TGFBR2, and TMED2 [13], were significantly downregulated after infection (Fig. 2D, E), particularly these results from the hematoxylin-eosin (HE) staining also demonstrated that alveolar cells and structures of the two dead patients were obviously damaged compared with the age-matched control group (Fig. 2F, G).

We further performed the functional enrichment analysis on all differentially expressed genes (DEGs) in AT1 and AT2 cells, and found that these DEGs were significantly enriched in energy electron chains, microtubule, response to virus, interferon response and neutrophil chemotaxis, and so on (Fig. 2H), suggesting that AT1 and AT2 cells can induce immune responses or antiviral activation signals to combat SARS-CoV-2 via releasing certain signaling molecules. Additionally, we found that the total frequency of lung cell communications was drastically reduced after infection (COVID-19: 3275 vs Health: 6973) (Fig. S1A, B and Table S3), which may be caused by these dead lung cells. Of note, these cell types with the highest frequency of cell communication among AT2/AT1 cells were MKI67+, End and basal cells in the healthy group, but the infection group was Macro, Neu, and Mono cells (Figs. 2I, J and S1C, D). Especially, significantly altered receptor and ligand genes in AT1 and AT2 cells were mainly enriched in myeloid cell migration, granulocyte migration, monocyte migration, and chemotaxis (Fig. 2K). Further, we analyzed the independent respiratory single-cell data from patients with lethal COVID-19 [14], and found that many alveolar-related genes were expression dysregulation, and the cell communication frequency of AT1/AT2 with myeloid cells was obviously increased, as well as the antiviral signal was significantly activated in these patients (Fig. S2), which were agreement with our conclusions.

### Ligand and receptor-mediated lung immune activation and inflammation

**Fig. 3:**
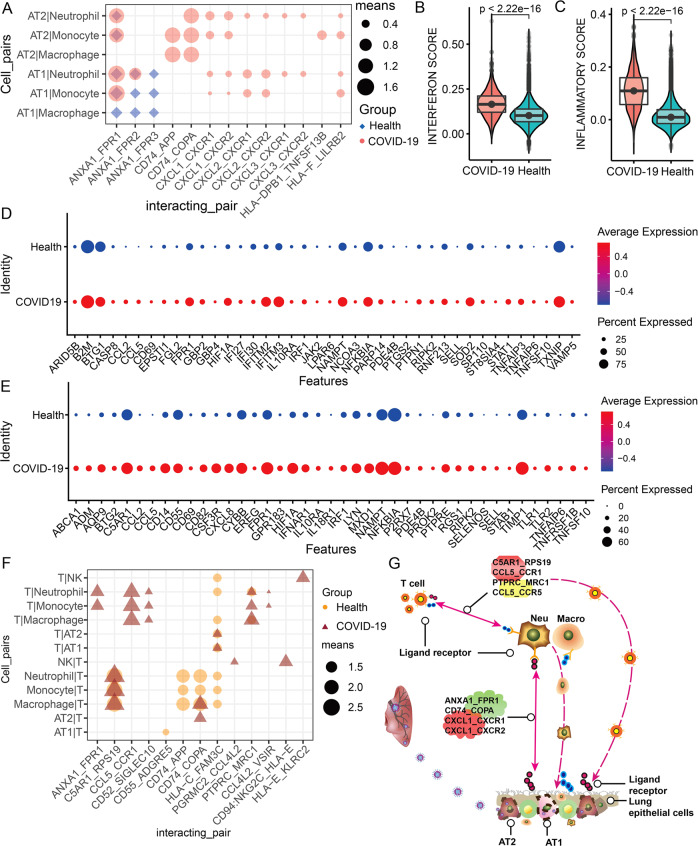
AT1/AT2 cells recruit and activate immune cells via the cellular communication. **A** The ligand and receptor molecules are significantly changed during the cell communication between AT1/AT2 and myeloid cells. **B**, **C** The interferon response and inflammatory gene set scores of myeloid cells (neutrophils/monocytes/macrophages in healthy groups and COVID-19 patients. **D**, **E** Significantly differentially expressed interferon response and inflammatory genes in lung myeloid cells of healthy groups and COVID-19 patients. **F** The receptor and ligand molecules that are significantly changed during the cell communication between AT1/AT2 and T/NK cells. **G** Schematic diagram of AT1/AT2 cells recruiting myeloid cells and T cells through specific ligands and receptors. The “means” in the legend represent the activity of the cell communication molecule (ligand_receptor). Only ligands and receptors that are specific or significantly changed (fold change>1.2, *p* < 0.01) between groups are displayed.

Herein, we further explored how infected alveolar target cells induce immune cell aggregation and immune activation. Interestingly, our results demonstrated that interaction scores of some paired ligands and receptors (e.g., ANXA1_FPR1, CD74_APP, CD74_COPA, CXCL1_CXCR1, CXCL2_CXCR2, and HLA-F_LILRB2) were significantly increased after infection (Fig. 3A). Of note, previous studies have indicated that the CXCLs_CXCRs family as known chemokines can induce the recruitment of Neu and Mono [15], and the ANXA1_FPR1 is related to the increase of myeloid cells of COVID-19 patients [16], as well as both CD74_COPA and CD74_APP can serve as inflammatory recruitment signals, particularly COPA is also reported to be involved in the occurrence of autoimmune interstitial lung and lipopneumonia [17, 18]. Taken together, we suggested that these above-paired ligands and receptors may participate in the recruitment of myeloid cells. Moreover, our findings showed that these myeloid cells (Neu and Mono/Macro) recruited by AT/AT2 cells had higher interferon response and inflammatory gene set scores (Figs. 3B, C and S3A, B), specifically interferon-related genes (e.g., *ARID5B*, *B2M*, *BTG1*, *IFITM2*, *IRF1*, *JAK2*, *STAT1,* and *TXNIP*) (Figs. 3D and S3C) as well as inflammation-related genes (e.g., *C5AR1*, *CD55*, *CSF3R*, *CXCL8*, *HIF1A*, *NAMPT*, *NFKB1A, and TLR2*) (Figs. 3E and S3D) were more higher expressed in myeloid cells, indicating that myeloid cells participate in immune response to SARS-CoV-2 infection.

Remarkably, few distinct ligand and receptor signals were observed in AT1/AT2 cells and T cells after infection (Fig. 3F), implying that the recruitment of T cells in the lung may be indirectly related to AT1/AT2 cells. In contrast, some paired ligands and receptors, such as ANXA1_FPR1, C5AR1_RPS19, CCL5_CCR1, and PTPRC_MRC1, were significantly activated after infection (Fig. 3F), which may mediate the communication among T cells and myeloid cells. Previous studies have also revealed that the combination of CCL5 and CCR1 can induce the recruitment of T cells to inflammation and viral sites [19–21]. Taken together, our work seems to indicate that myeloid cells may have obvious duality role in the infected lungs. On the one hand, these myeloid cells can be recruited to AT1/AT2 cells through dysregulated ligands and receptors, and become the source of the inflammation storm in the lungs (Figs. 3E, G, and S3D). On the other hand, these immune-activated myeloid cells can further induce T lymphocytes to participate in the inhibition of SARS-CoV-2 (Fig. 3F, G).

### Increased fibrosis and mucus accumulation in lung inflammatory environment

**Fig. 4:**
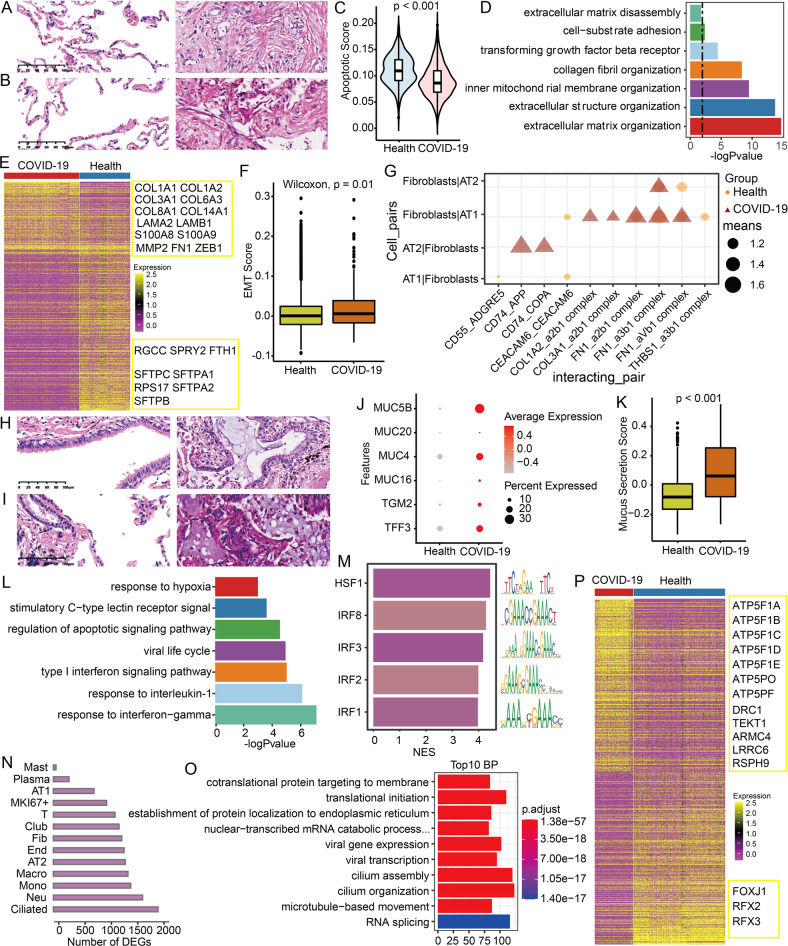
Increased fibrosis and mucus accumulation in lung inflammatory environment. **A**, **B** HE staining showed increased pulmonary fibrosis in COVID-19 group compared with control group. On the left are two controls (C1, C2) and on the right are two dead patients (P1, P2). **C** The difference in apoptosis scores of fibroblasts between healthy and COVID-19 groups. **D** Differentially expressed genes (DEGs) of fibroblasts are enriched in the biological processes related to fibrosis. **E** Expression changes of fibrosis-related genes in healthy and COVID-19 groups. **F** The difference in EMT scores between healthy and COVID-19 groups. **G** Significantly changed ligand and receptor during the communication between AT1/AT2 cells and fibroblasts. **H**, **I** HE staining showed increased mucus accumulation in COVID-19 compared with control. **J** Expression changes of genes related to mucus formation and mucus consistency in healthy and COVID-19 groups. **K** The difference in EMT scores between healthy and COVID-19 groups. **L** The DEGs of club cells are enriched in biological processes related to antiviral and inflammation. **M** Top transcription factor enriched from DEGs in club cells. **N** The number of DEGs in different cells of the lung. **O** The top 10 enriched biological process of DEGs in ciliated cells. **P** Expression changes of genes related to cilia function in healthy groups and COVID-19 patients.

Epithelial-mesenchymal transition (EMT) can induce fibroblasts for endless wound repair after trauma and inflammatory injury [22], so we further explore this issue here. Interestingly, our results also showed a significant lung fibrosis increase in the two dead COVID-19 patients from the HE staining (Fig. 4A, B), and a significantly reduced apoptosis score of fibroblasts compared with the control (Fig. 4C). Further, these DEGs in fibroblasts were significantly enriched in extracellular matrix decomposition, cell adhesion and extracellular matrix assembly (Fig. 4D). These above results suggested that the cytoskeleton and cell morphology may have been altered after SARS-CoV-2 infection. Especially, we found that collagen proteins COL1A1, COL1A2, COL3A1), laminin proteins (LAMA2, LAMB1), inflammatory proteins (S100A8, S100A9), the matrix metalloproteinase MMP2, fibronectin FN1, and regulator ZEB1 were significantly upregulated in the fibroblasts of COVID-19 patients (Fig. 4E), which have been proved to be related to the increase of EMT-mediated fibrosis [22–24]. Of note, these top-downregulated genes in COVID-19 fibroblasts are mainly members of the SFTP family, which have been proved to be markers of alveolar structure and lung epithelial cells [13]. Meanwhile, *RGCC* (Regulator of Cell Cycle), *SPRY2* (Sprouty RTK Signaling Antagonist 2), and *FTH1* (Ferritin Heavy Chain 1) were also significantly downregulated, and they have been verified to negatively regulate fibroblast growth factor or proliferation [25–27]. These results further revealed that fibrosis of COVID-19 patients can be induced by EMT accompanied by a significantly increased EMT score (Fig. 4F), and the decrease of epithelial cell marker genes *MAL2*, *CLND4*, and *TJP1*, as well as the increase of *VIM* and *CDH2* of mesenchymal cell (Fig. S4A, B). Previous studies have also demonstrated that the activation of COL1A2_a2b1, COL3A1_a2b1, and FN1_a3b1 signals could promote the EMT and pulmonary fibrosis [22, 28]. Together, our present work suggested that some paired receptors and ligands, such as CD74_APP, CD74_COPA, COL1A2_a2b1, COL3A1_a2b1, and FN1_a3b1, not only may mediate the cell communication between AT2 cells and fibroblasts, but also may facilitate the increase of lung fibrosis EMT-mediated by the inflammation (Fig. 4G).

In addition to pulmonary fibrosis, our HE staining results revealed significant mucus increase in the lungs of COVID-19 patients (Fig. 4H, I), which was consistent with the increase of the proportion of club cells that produce mucus (Fig. 2C). Particularly, some mucus components (e.g., MUC5B and MUC4) and genes related to mucus viscosity (e.g., *TGM2* and *TFF3*) were significantly upregulated in COVID19 patients (Fig. 4J), as well as the mucus secretion score of club cells was significantly increased (Fig. 4K). These results seemed to indicate that SARS-CoV-2 infection can improve the ratio of club cells and promote mucus production. Interestingly, our results also demonstrated that these DEGs in club cells were mainly enriched in hypoxia, C-type lectin, virus life cycle, interferon response process, and so on (Fig. 4L), and HSF1, IRF8, IRF3, IRF2, and IRF1 were the top 5 enriched transcription factors (Fig. 4M), which have been reported to be associated with inflammation and immune responses [29, 30].

Of note, most DEGs in ciliated cells were significantly enriched in protein translation, microtubule movement, cilia assembly, cilia structure, and so on (Fig. 4N, O), implying that the structure and movement of the cilia may have been disrupted after infection. Especially, some cilia production-related regulators (e.g., FOXJ1, RFX2, and RFX3) and cilia structure, cilia movement and microtubule bundle movement-related genes (e.g., *DRC1*, *TEKT1*, *ARMC4*, *LRRC6*, *RSPH9*) were significantly disordered after infection (Fig. 4P), which were consistent with these previous reports [31–36]. Previous studies also indicated that the frequency of cilia beating could be destroyed and the secretion of airway mucin could be increased thousands of times under the stimulation with high levels of extracellular ATP [31, 37]. Interestingly, we also found that ATP synthesis-related genes (e.g., *ATP5F1A*, *ATP5F1B*, *ATP5F1C*, *ATP5F1D*, *ATP5F1E*) were significantly increased in COVID-19 patients (Figs. 4P and S5). Collectively, our results revealed that SARS-CoV-2 infection-induced inflammation may severely disrupt the homeostasis of ciliated cells, thereby resulting in the impaired function for cleaning.

#### Single-cell atlas and ligand-receptor changes in the blood of COVID-19 patients

**Fig. 5:**
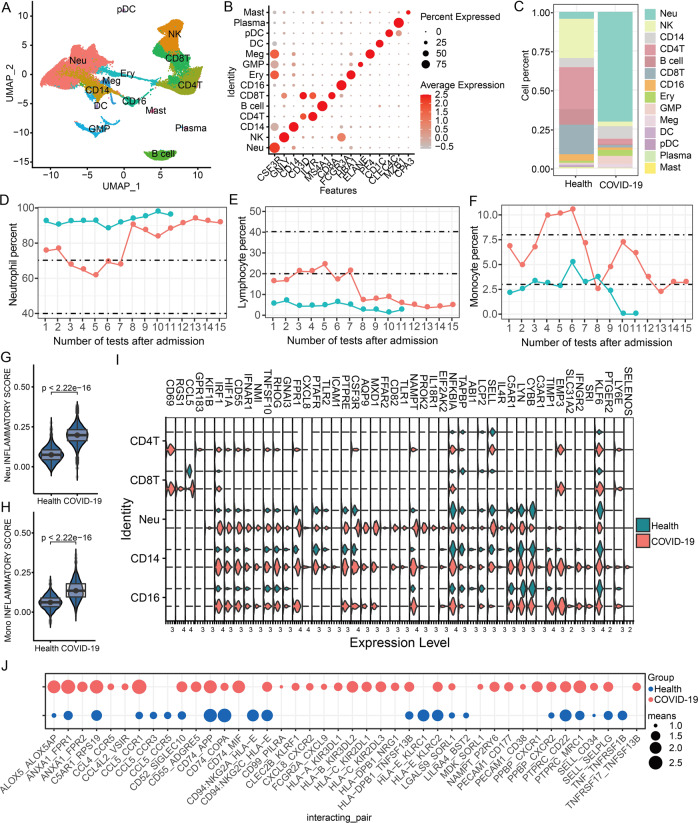
Single-cell map of blood and its molecular changes. **A** The UMAP cluster map shows the clustering results of cell types in the blood of healthy and COVID-19 patients. **B** The expression levels of classical marker genes corresponding to different cell types in the blood. C: The proportion of different cell types in the blood of healthy groups and COVID-19 patients. **D**, **F** Detect the ratio of neutrophils, lymphocytes, and monocytes in the blood of two COVID-19 patients. Blood was collected multiple times for testing before death. The dashed lines represent the normal reference range. **G**, **H** Differences in inflammatory gene set scores between neutrophils and monocytes in healthy groups and COVID-19 patients. **I** Significantly differentially expressed inflammation-related genes in blood neutrophils and monocytes in healthy groups and COVID-19 patients. **J** Significantly changed ligand and receptor molecules in the blood of healthy group and COVID-19 patients. The “means” in the legend represent the activity of the cell communication molecule (ligand_receptor).

Based on the single-cell atlas of the blood, we identified multiple kinds of myeloid cells including Neu (CSF3R+), CD14 monocyte (CD14+), CD16 monocyte (FCGR3A+), dendritic cells (DC) (CD1C+), plasmacytoid dendritic cells (pDC) (CLEC4C+), and mast cells (CPA3+) (Fig. 5A, B), and numerous immune cells including B cell (MS4A1+), plasma cell (MZB1+), CD4 T (CD3D+, IL7R+), CD8 T (CD3D+, CD8A+), and NK (Fig. 5A, B), as well as other granulocyte-macrophage progenitor (GMP) (ELANE+), megakaryocytes (Meg) (PF4+), and erythroid cells (Ery) (HBA1+) (Fig. 5A, B). Of note, these significantly increased cell types were Neu and GMP, but these decreased cell types were T cell and NK cell in blood of COVID-19 patients (Fig. 5C), which were agreement with the multiple blood test results that Neu was significantly higher than the normal value after infection (Fig. 5D), but the lymphocyte ratio was opposite (Fig. 5E). Interestingly, although the laboratory indicators (percentage of neutrophils, percentage of lymphocytes, C-reactive protein, IL-6) of the two COVID-19 patients were almost uniformly significantly higher or lower than the normal range (Figs. 1H, I and 5D, E), their subtle differences may reflect the rate of disease progression of the patients. More importantly, these test results of the two COVID-19 patients before death were consistent, suggesting that they may have experienced a consistent inflammatory storm and lymphopenia before death. The two COVID-19 patient’s monocytes also presented an abnormally disordered state (Fig. 5F), particularly both Neu and Mono cells in the blood of patients had a significantly increased inflammatory score (Fig. 5G–H), suggesting that they may have experienced active immunity and inflammatory storm before death. For example, multiple immune and inflammation-related genes, such as *C5AR1*, *CD55*, *CSF3R*, *CXCL8*, *FPR1, TLR1, HIF1A, IFNAR1A, IRF1,* and *NFKB1A*, were significantly upregulated in the blood immune cells of COVID-19 patients (Figs. 5I and S6A, C), which was similar to the lungs (Fig. 3D, E).

Different from the decrease in blood, these lymphocyte cells (e.g., T cells and NK cells) were significantly increased in the lungs of COVID-19 patients (Fig. 2C). This phenomenon may be caused by the migration of blood immune cells to the lungs induced by SARS-CoV-2 infection. Remarkably, ACE2 and TMPRSS2 are extremely important for SARS-CoV-2 to enter cells [38], but we hardly detected expressions of ACE2 and TMPRSS2 in the blood of COVID-19 patients (Fig. S7A, B). Why and how do immune responses and inflammation in the blood of COVID-19 patients be induced without ACE2 and TMPRSS2? Interestingly, we found that C5AR1 and FPR1 were highly expressed in myeloid cells of the patient blood (Fig. 5I), particularly C5AR1 and FPR1 have been proved to be cell communication molecules that can transmit inflammatory signals [39–42]. So we further investigated which signal molecules could cause inflammation of the blood. As shown in Fig. S7C, those significantly altered ligands and receptors in the blood were mainly enriched in migration, proliferation, and cell adhesion of leukocytes, myeloid cells, and other processes, suggesting that these paired ligands and receptors may be responsible for inflammation and cell migrations. Moreover, we found some significantly changed ligands and receptors (ALOX5_ALOX5AP, ANXA1_FPR1, ANXA1_FPR2, C5AR1_RPS19, CCL5_CCR1, and CD74_MIF) in the blood (Figs. 5J and S6D), which are similar to these results from lung (Fig. 3). Especially, the enrichment of a set of activated HLA family ligands also suggested that SARS-CoV-2 infection may activate antigen presentation and antiviral responses in blood (Fig. 5J). Taken together, our study seemed to imply that certain specific receptors and ligands in the blood can act as cell communication molecules in response to inflammatory signals from the lung, subsequently induce immune responses, and promote immune cells (e.g., T cells and neutrophils) to migrate to the lungs.

### Single-cell atlas and ligand-receptor changes in the bone marrow of COVID-19 patients

**Fig. 6:**
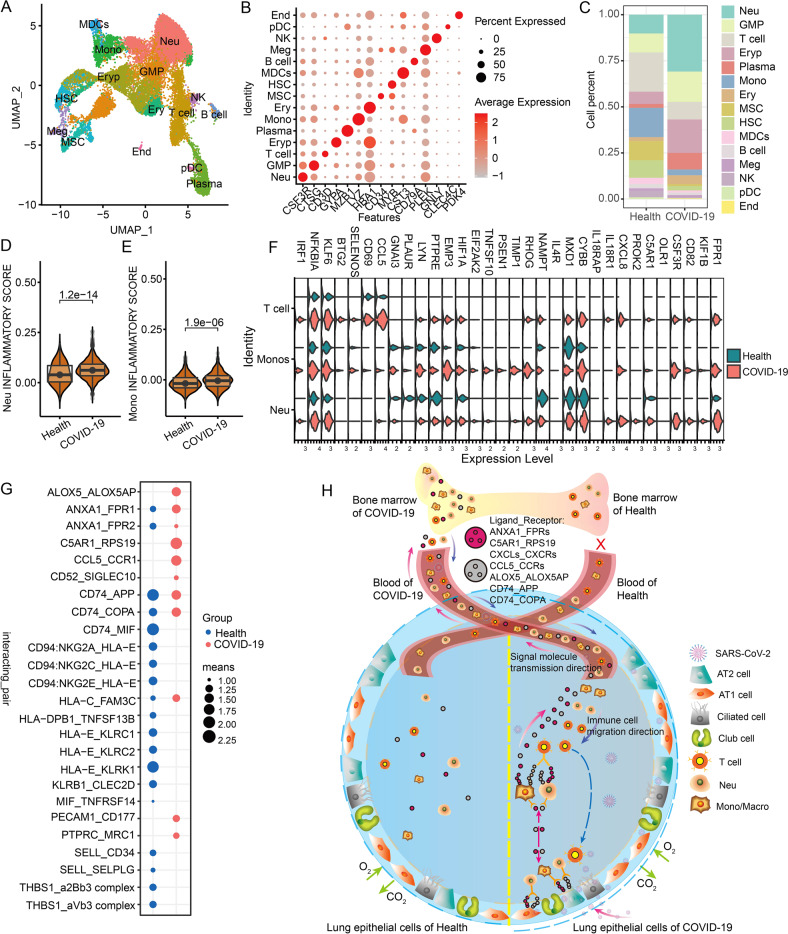
Single-cell map of bone marrow and its molecular changes. **A** The UMAP cluster map shows the clustering results of the bone marrow cell types of healthy and COVID-19 patients. **B** The expression levels of classical marker genes corresponding to different cell types in the bone marrow. **C** The proportion of different cell types in the bone marrow of the healthy group and COVID-19 patients. **D**, **E** Differences in the inflammatory gene set scores of bone marrow neutrophils and monocytes in the healthy group and COVID-19 patients. **F** Significantly differentially expressed inflammation-related genes of bone marrow neutrophils and monocytes in healthy groups and COVID-19 patients. **G** Receptor and ligand molecules undergo significant changes between different cell types in the bone marrow. The “means” in the legend represent the activity of the cell communication molecule (ligand_receptor). **H** Ligand/receptor-mediated immune cell migration and antiviral response patterns of lung, blood, and bone marrow. The purple arrows represent the transmission of signaling molecules (ligands/receptors), and the blue arrows represent the migration direction of immune cells.

To further reveal whether the bone marrow responds to SARS-CoV-2 infection and is responsible for the abundance change of immune cells, we further analyzed the single-cell data from the two dead COVID-19 patients. In the bone marrow, we identified common erythroid progenitor cells (Eryp) (GYPA+), Ery (HBA1+), GMP (CTSG+), hematopoietic stem cells (HSC) (MYB+) and mesenchymal stem cells (MSC) (CD34+), and many myeloid cells such as Neu (CSF3R+), monocyte-derived dendritic cells (MDCs) (CST3+), Mono (LYZ+), pDC (CLEC4C+), as well as T cells (CD3D+), NK cells (GNLY+), B cells (CD79A+), plasma cells (MZB1+), and so on (Fig. 6A, B). Furthermore, we identified Meg (PLEK+) and a small number of End cells (PDK4+) (Fig. 6A, B). Among all cell types, the proportion of Neu, GMP, and Plasma cells increased obviously after SARS-CoV-2 infection (Fig. 6C). Remarkably, Neu in the bone marrow of COVID-19 patients also increased significantly (Fig. 6C), which is similar with the blood and lungs (Figs. 5C and 2C). Whilst the proportion of T cells and Mono in the bone marrow of COVID-19 patients reduced significantly (Fig. 6C), which is similar with the blood (Fig. 5C).

Herein, we further evaluated the inflammatory scores of Neu and Mono cells in the bone marrow, finding that the inflammatory scores of COVID-19 patients were significantly higher than those of normal people (Fig. 6D, E). Particularly, many inflammation genes, such as *C5AR1*, *CD55*, *CSF3R*, *CXCL8*, *FPR1*, *KLF6*, and *NFKB1A*, were significantly upregulated in Neu and Mono cells of the bone marrow of COVID-19 patients (Fig. 6F). It is worth noting that the inflammatory scores in the bone marrow were not as high as those in the lungs and blood because the number of inflammatory genes were significantly lower than the lungs and blood of the COVID-19 patients (Figs. 3, 5, and 6). Especially, expressions of ACE2 and TMPRSS2 almost none were detected in the bone marrow (Fig. S8A, B), suggesting that the immune and inflammatory response of the myeloid cells of bone marrow might be caused by signal transmission between distinct cells or tissues, rather than SARS-CoV-2 directly infects the bone marrow. Interestingly, the functional enrichment analysis revealed that signaling molecules of the bone marrow cells were widely enriched in the leukocyte and myeloid cell migration, activation, proliferation, and chemotaxis (Fig. S8C). Therefore, we focused on these significantly changed ligands and receptors in the bone marrow, and found that ALOX5_ALOX5AP, ANXA1_FPR1, CCL5_RPS19, CCL5_CCR1, CD74_COPA, and so on (Fig. 6G) also existed in the lung and the blood (Figs. 3 and 5). Together, our study seemed to indicate that after the lungs were infected by SARS-CoV-2, these ligands and receptors shared in the blood and the bone marrow may respond to the immune and inflammatory signaling from the lung, and induce the activation of immune cells and migrate them into the lungs.

## Discussion

Systemic inflammatory storm and lymphopenia caused by SARS-CoV-2 infection are important factors of resulting in the death of COVID-19 patients. ACE2 and TMPRSS2 have been proved to be required for SARS-CoV-2 entry, but their expressions are hardly detected in cells of multiple tissues, such as blood and bone marrow (Figs. S7 and S8). Therefore, our goal was to decipher how locally infected AT1 and AT2 cells contribute to the inflammatory microenvironment of the lung and the systemic immune response. In this paper, we find that ligands and receptors shared by different tissues may be the driving force of causing inflammatory storm and immune cell migration (Fig. 6H).

Our present work revealed that SARS-CoV-2 infected the alveolar target cells AT1/AT2 to result in immune activation and release a large number of inflammation-related ligands and receptors (such as ANXA1_FPRs, CXCLs_CXCRs, CD74_APP, CD74_COPA, CCL5_CCRs) (Figs. 3 and 6H), which could further bind to their receptors on free myeloid cells in the lung cavity to rapidly induce their enrichment to the infection site. For example, the ligand ANXA1 and receptor FPRs can promote the maturation neutrophil cells and macrophages and migrate them into the damaged lung tissue [43]. On the one hand, these recruited Neu, Mono/Macro cells not only may inhibit the SARS-CoV-2 virus and clear damaged cells, but also may further recruit T cells to the infection site to increase the inhibition of SARS-CoV-2 through certain ligand and receptor pairs (e.g., ALOX5_ALOX5AP, C5AR1_RPS19, CCL5_CCR1) (Figs. 3 and 6H) [19–21, 43, 44]. On the other hand, the binding of inflammation-related ligands to their receptors can induce myeloid cells to release many inflammatory genes, resulting in an inflammatory microenvironment in the lungs (Figs. 3 and 6H). For instance, C5AR1 can act as an upstream signal of Toll-like pathways (NFKB1, TLR1, TLR2, etc.) [44–46] and CSF3R can act as a receptor to promote the activation of granulocytes and macrophages [47]. Particularly, CSF3R can also further activate these downstream inflammatory cytokines, such as TNFRA family members (TNFAIP6, TNFRSF1B, TNFSF10) and IL family members (IL18R1, IL1B, IL7R) [48, 49], which was agreement with our results (Figs. 3D, E and S3). Of note, the inflammatory microenvironment in the lungs may provide soil for EMT and pulmonary fibrosis to lead to lung obstruction and poor breathing by promoting the production of mucus by club cells and disrupting the clearing function of ciliated cells [22–24, 31], which was also agreement with our findings (Figs. 4, 6H, S4 and S5).

In our work, we found that multiple activated ligand and receptor pairs, such as ANXA1_FPRs, CXCLs_CXCRs, CCLs_CCRs, C5AR1_RPS19, CD74_COPA, and ALOX5_ALOX5AP, were shared among lung, blood and bone marrow of patients (Fig. 6H), implying that they may be involved in the recruitment and migration of immune cells across tissues. For example, ALOX5 and ALOX5AP were shown to be related to the respiratory system [50]. The upregulated C5AR1 led to excessive inflammation and coagulation complement system disorders [40]. CD74 promoted MIF-mediated inflammation via interacting with MIF on the membrane surface of alveolar macrophages to activate the p44/p43 MAPK signaling pathway and induce the accumulation of neutrophils in the alveolar cavity [51]. The upregulated expression of CXCL9 and CXL10 can respectively function as CXCR3 ligands on T cells to ensure migration of CD8 + T cells to the specifically peripheral organs and kill virus-containing cells [52]. The upregulated expression of CXCR3 allowed T cells with circulating effectors to home to inflamed lymph nodes [53]. Additionally, CXCL10 and CCL2 were highly expressed in patients with SASR-CoV infection, especially their upregulation expressions can inhibit the development of hematopoietic precursor cells and cause lymphopenia in patients infected with SASR-CoV [54–57]. Interestingly, our present results also revealed that the multiple members of CXCLs and CXCRs families are significantly upregulated in the lungs, blood, and bone marrow of COVID-19 patients.

Taken together all functions of these ligands and receptors, our results suggested that these inflammatory signaling molecules may originate from AT1 and AT2 cells of the infected lung, especially they circulated into the blood and the bone marrow to further induce immune cell migration and inflammatory gene storm across different tissues (Fig. 6H). On the one hand, T cells in the blood and the bone marrow can be recruited to infection sites in the lungs, thereby leading to a decrease of T cells in the blood and the bone marrow (Figs. 5 and 6). Interestingly, although neutrophils also migrate to the lungs, they are still abundant in blood and bone marrow (Figs. 2, 5, and 6), which may be attributed to the imbalance of HSC differentiation in the hematopoietic system, resulting in increased differentiation of myeloid progenitor cells and decreased lymphoid progenitor cells [58, 59]. On the other hand, continuous inflammatory signals induce large amounts of inflammatory gene expressions in different tissues to cause a systemic inflammatory storm (Figs. 3, 5, 6H, S3, and S6).

In conclusion, these shared inflammatory ligands can enter the circulatory system and bind to their receptors across different tissues to transmit inflammatory and antiviral signals, thereby promoting systemic immune responses and even inflammatory storms (Fig. 6H). It should be noted that although the early autopsy samples of COVID-19 patients are limited, these main conclusions of our work are supported by the single-cell validation set (Figs. S2–S6). In addition, these ligands and receptors deduced from the single-cell transcriptome presented only changes in mRNA levels, but the lung proteome data of COVID-19 patients basically demonstrated that these activated ligands and receptors were upregulated to varying degrees (Fig. S9). Therefore, these identified inflammatory ligands and receptors may be expected to be potential intervention targets for the treatment of severe COVID-19 patients. For example, the combination of C5AR1 and RPS19 mainly activate the coagulation complement system [39, 40], and C5AR1 monoclonal antibody is also considered to be a target for the treatment of coagulopathy caused by SARS-CoV-2 [60, 61].

## Materials and methods

### Ethical approval and consent to participate

This study was conducted in accordance with legitimate ethical principles. We obtained prior approval from the Ministry of Education of China, Southwest Hospital, Third Military Medical University (Army Medical University), and Jinling Hospital, Nanjing University School of Medicine. COVID-19 samples were obtained with approval from the Research Ethics Committee of Huoshenshan Hospital. All samples used in this study obtained written informed consent from donors or their relatives.

### Sample collection

The lung, blood, and bone marrow tissues of two COVID-19 patients were obtained as described in previous literature from Huoshenshan Hospital, Wuhan, China [1, 2]. The 77-year-old patient 1 suffered from malignant tumor, type 2 diabetes, Klebsiella infection, fever, and pulmonary ground glass. The 81-year-old patient 2 had hypertension, coronary heart disease, Alzheimer’s disease, and also had unknown bacterial infection, headache, weakness, and ground-glass lung symptoms (Table S1). Both patients were treated with arbidol antiviral drugs. A small number of fresh autopsy tissues of COVID-19 patients were immediately used for single-cell transcriptome sequencing and most of the remaining formalin fixed, paraffin embedded (FFPE) postmortem specimens were used for viral RNA detection (Table S2), tissue staining or morphological analysis [1, 2]. The normal lung tissue of the age-matched patients who died of sudden cardiac death or cancer was taken as the control to compare IHC and HE.

### Tissue staining

Hematoxylin and eosin (H&E) and immunohistochemistry (IHC) staining were performed according to the standard procedures described previously [1, 2]. The sections were incubated with the primary antibody overnight at 4 °C. Pathological lesions and SARS-CoV-2 spike protein were independently quantified by two pathologists. For each patient, the calculation required at least 20 randomly selected 100x microscopic fields.

### Serological testing

The blood cell count of patients was tested using Mindray’s automatic blood cell analyzer (BC-5390CRP). Overall 2 ml EDTA-K2 anticoagulated venous blood needed to be put into the sample holder for testing. The instrument can detect lymphocytes, monocytes, and eosinophils in basophils and neutrophils through laser light scattering and flow cytometry in the DIFF laser channel.

### Tissue dissociation and single-cell suspension preparation

Fresh tissue samples were washed with Hanks Balanced Salt Solution (HBSS) and minced into pieces. The pieces were digested in GEXSCOPE Tissue Dissociation Solution (Singleron Biotechnologies). A 40-µ sterile strainer was used to separate cells from cell debris and other impurities. The cells were centrifuged, and cell pellets were resuspended in PBS (HyClone). To remove red blood cells, GEXSCOPE Red Blood Cell Lysis Buffer (Singleron Biotechnologies) was added to the cell suspension. The mixture was then centrifuged and resuspended in PBS. Cells were counted with TC20 automated cell counter (Bio-Rad).

### Single-cell RNA sequencing library preparation and generation of gene expression

The concentration of single-cell suspension was adjusted to 1×10^5^ cells/mL in PBS. Single-cell suspension was then loaded onto a microfluidic chip (GEXSCOPE Single Cell RNAseq Kit, Singleron Biotechnologies) and scRNA-seq libraries were constructed according to the manufacturer’s instructions. The resulting scRNA-seq libraries were sequenced on an Illumina HiSeq X10 instrument with 150 bp paired-end reads. Raw reads were processed to generate gene expression matrices by CeleScope software. We removed cells that had either lower than 200 or higher than 5000 expressed genes and cells with >30,000 UMIs and mitochondria content higher than 50%.

### Single-cell data analysis

We used Seurat package (version 3) to integrate single-cell data [62]. The CCA anchor algorithm was used to remove batch effects [62]. The top 2000 feature variables were used for subsequent dimensionality reduction. The *AddModuleScore()* of Seurat was used to evaluate the cell’s gene set score [62] and the gene set mainly came from msigdb R package. Wilcox test was used to calculate the significance of gene set scores. The CellPhoneDB software was used to analyze the interactions between cells and discover important receptor-ligand pairs after filtering out the combinations with low interaction scores (Table S3) [6].

### Differentially expressed gene analysis

The Seurat’s *Findmarker()* was used to find differentially expressed genes (DEGs) (*|logFC* | > 0.25 & *fdr* < 0.01). Gene ontology and KEGG (Kyoto Encyclopedia of Genes and Genomes) pathway enrichment analysis were completed by the clusterProfiler R package [63]. Using RcisTarget software to perform transcription factor enrichment analysis on DEGs.

## Supplementary information

Supplementary Figures

Supplementary Tables

reproducibility checklist

## Data Availability

The raw sequencing data of this work were deposited at GEO database (GSE154395). In addition, 8 public normal lung tissues (GSE122960) [7], 2 normal bone marrow (GSE116256) [8], and 6 normal blood tissues (GSE150728) [9] were also included (Table S4). The PBMC data of the validation set was from GSE149689 [64], and only severe and control samples were used. The lung single-cell data set of lethal COVID-19 was from GSE171524 [14] and the lung proteomics data were from the research of Wu et al. [65]. All data can request from the corresponding author XYX (xinyixia@nju.edu.cn).
