# Supplementary material for: Novel insight into the underlying dysregulation mechanisms of immune cell-to-cell communication by analyzing multitissue single-cell atlas of two COVID-19 patients
Source: Cell Death Dis. 2023 Apr 22;14(4):286. doi: 10.1038/s41419-023-05814-z (PMC10122452; doi:10.1038/s41419-023-05814-z)
Supplement: Supplementary file 1 — Supplementary Figures [file 41419_2023_5814_MOESM1_ESM.pdf]

Novel insight into the underlying dysregulation mechanisms of immune cell-to-cell communication by analyzing multi-tissue single-cell atlas of two COVID-19 patients

Shijie Qin[1,3#], Xiaohong Yao[2,11#], Weiwei Li[1#], Canbiao Wang[3#], Weijun Xu[1,4#], Zhenhua Gan[1,11#], Yang Yang[1], Aifang Zhong[5,11], Bin Wang[6,11], Zhicheng He[2,11], Jian Wu[1], Qiuyue Wu[1], Weijun Jiang[1], Ying Han[1], Fan Wang[1], Zhihua Wang[7,11], Yuehua Ke[8,11], Jun Zhao[1], Junyin Gao[9,11], Liang Qu[10,11], Ping Jin[3], Miao Guan[3]*, Xinyi Xia[1,11]*, Xiuwu Bian[2,11]*

[1]Institute of Laboratory Medicine, Jinling Hospital, Nanjing University School of Medicine, 210002 Nanjing, Jiangsu, China.

[2]Institute of Pathology, Key Laboratory of Tumor Immunopathology, Ministry of Education of China, Southwest Hospital, Third Military Medical University (Army Medical University), 400038 Chongqing, China.

[3]Laboratory for Comparative Genomics and Bioinformatics, College of Life Science, Nanjing Normal University, 210046 Nanjing, Jiangsu, China.

[4]Department of Gastroenterology, Jinling Hospital, Nanjing University School of Medicine, 210002 Nanjing, Jiangsu, China.

[5]Medical Technical Support Division, the 904th Hospital, 213003 Changzhou, Jiangsu, China.

[6]Department of Gastroenterology, Daping Hospital, Third Military Medical University (Army Medical University), 400038 Chongqing, China

[7]Department of Laboratory Medicine & Blood Transfusion, the 907th Hospital, 350702 Nanping, Fujian, China.

[8]Center for Disease Control and Prevention of PLA, Beijing, China.

[9]Pulmonary and Critical Care Medicine, Yancheng No.1 People's Hospital, 224000 Yancheng, Jiangsu, China.

[10]Department of Laboratory Medicine, 920 Hospital of the Joint Service Support Force of the Chinese People's Liberation Army, 650032 Kunming, Yunnan, China.

[11]Joint Expert Group for COVID-19, Department of Laboratory Medicine & Blood Transfusion, Wuhan Huoshenshan Hospital, 430100 Wuhan, Hubei, China.

# These authors contributed equally

***Corresponding Authors:** bianxiuwu@263.net, xinyixia@nju.edu.cn or 08326@njnu.edu.cn

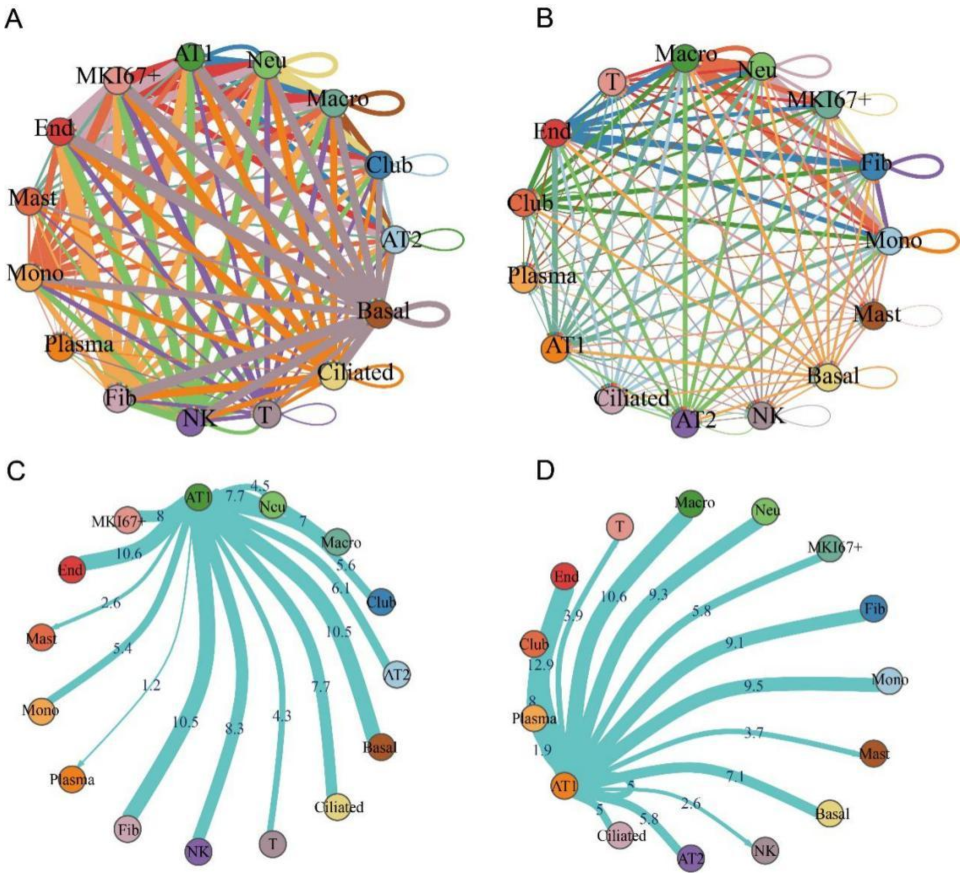

Fig S1. Cell communication analysis between COVID-19 and healthy groups.

A~B: The frequency of cell communication between different cell types in the healthy group and the COVID-19 group. C~D: The frequency of cell communication between AT1 cells and other cell types in the healthy group and the COVID-19 group. The thicker the line, the higher the communication frequency, the thinner the line, the lower the communication frequency

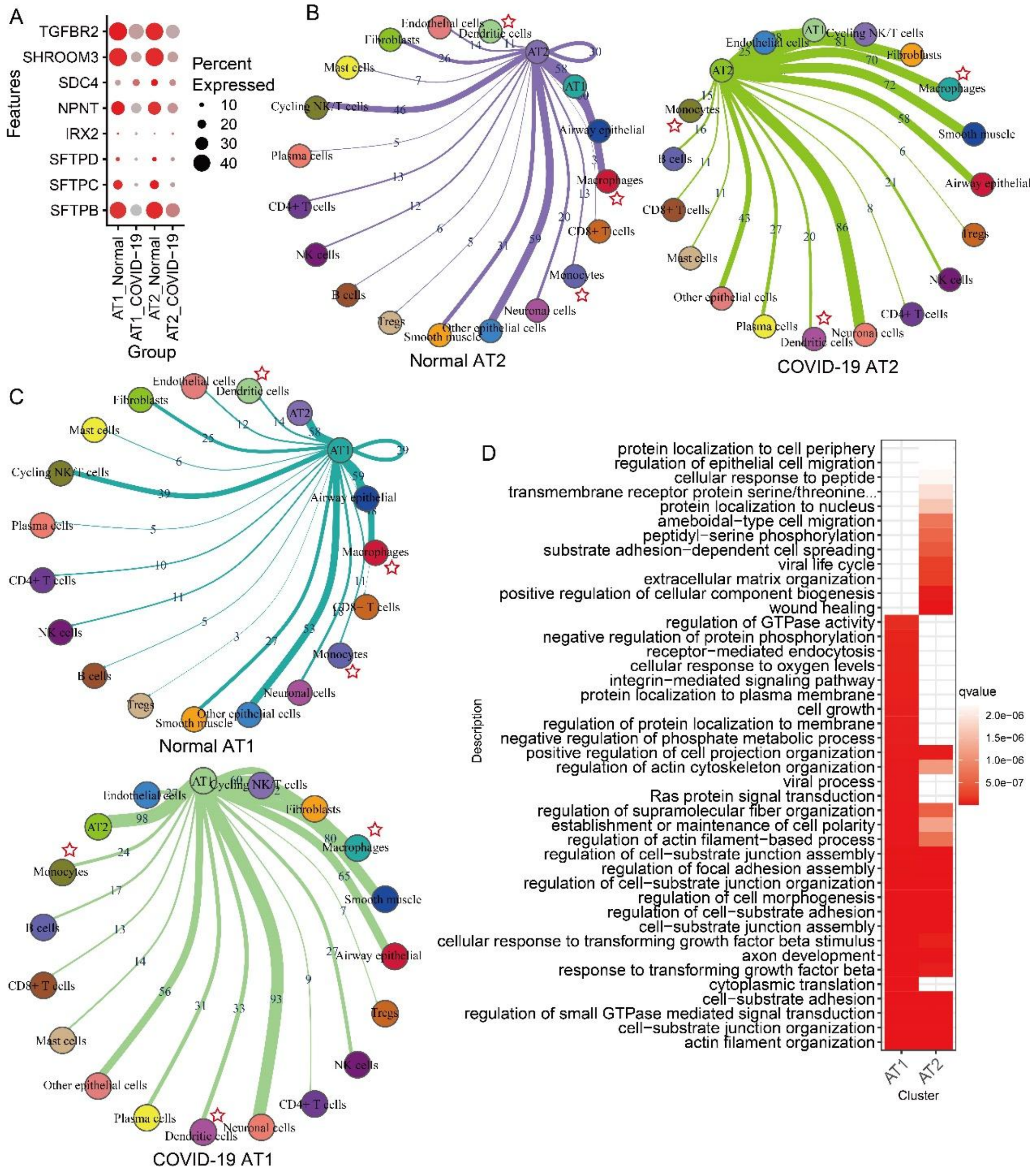

Fig S2. Alveolar gene and cell communication analysis in a lethal COVID-19 cohort.

A: Changes in alveolar-related gene expression levels in COVID-19 and normal groups. B: The communication frequency of AT2 cells with other cells was in the normal group and the COVID-19 group. C: The communication frequency of AT1 cells with other cells was in the normal group and the COVID-19 group. The red asterisk represents myeloid cells with increased communication with AT1 and AT2 cells. D: Enrichment analysis of differential genes between AT1 and AT2 cells in the lethal COVID-19 cohort. See Table S3 for complete cell communication ligand and receptor pairs. In general, the results in the fatal COVID-19 cohort also basically support the increased frequency of cell communication between alveolar cells and myeloid cells, and are immune-activated.The cell annotation of the fatal COVID-19 cohort was derived from the original data uploaded by the authors.

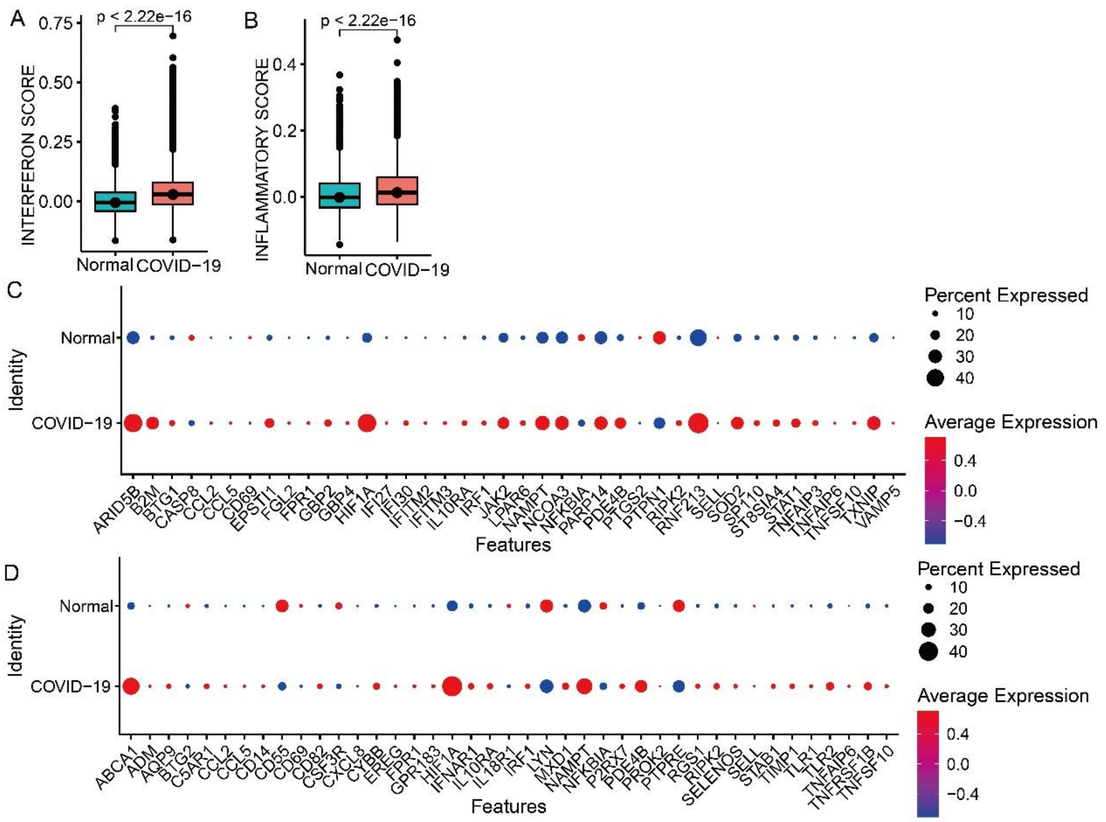

Fig S3. Interferon and inflammatory response analysis of lethal COVID-19 cohort.

A: The activity difference of interferon response gene set between COVID-19 patients and control group. B: The activity difference of inflammatory response gene set between COVID-19 patients and control group. C: The expression level of interferon related genes in COVID-19 patients and control group. D: The expression level of inflammation-related genes in COVID-19 patients and control group. Overall, the results in the lethal COVID-19 cohort also basically support the up-regulated interferon and inflammatory signals, as well as up-regulated genes in our data. The cell annotation of the fatal COVID-19 cohort was derived from the original data uploaded by the authors.

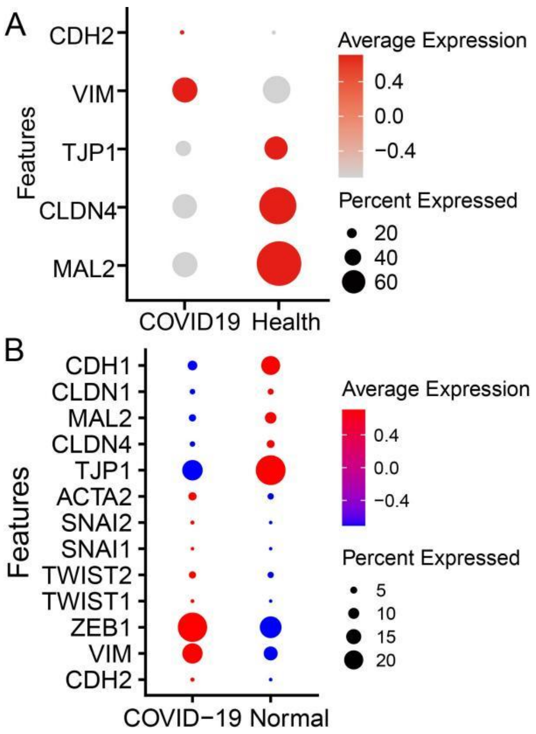

Fig S4. Markers involved in epithelial-mesenchymal transition (EMT).

A: Differences in the expression of EMT markers between the two dead patients and the healthy control group. A: Differences in the expression of EMT markers in the lethal COVID-19 cohort. The expression changes of epithelial cell markers MAL2, CLDN4, TJP1, CDH1, CLDN1 and mesenchymal cell marker VIM, CDH2, ZEB1, TWIST1, TWIST2, SNAI1, SNAI2, ACAT2 in healthy groups and COVID-19 patients. Our data results are basically consistent with those in the fatal COVID-19 cohort. The cell annotation of the fatal COVID-19 cohort was derived from the original data uploaded by the authors.

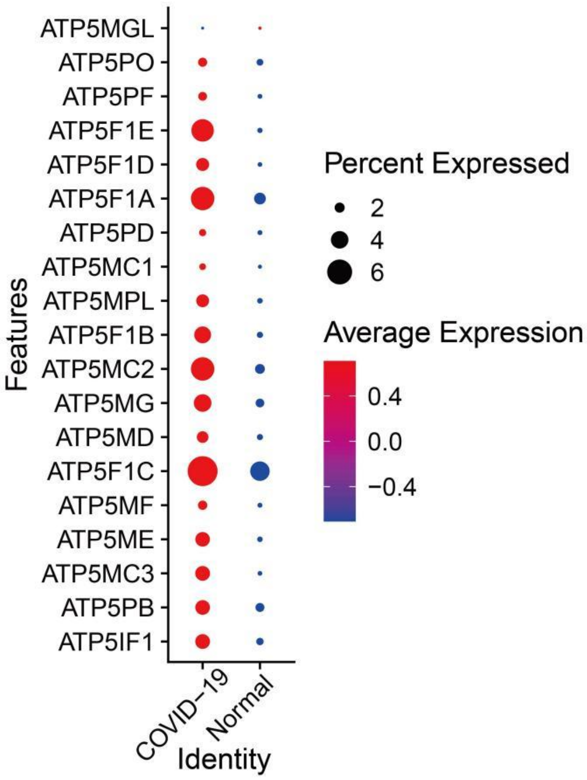

Fig S5. Changes in ATP-related gene expression in Airway ciliated cells in a lethal COVID-19 cohort.

Almost all of these ATP-related genes are significantly up-regulated after reinfection, which is consistent with the results shown in the manuscript.The cell annotation of the fatal COVID-19 cohort was derived from the original data uploaded by the authors.

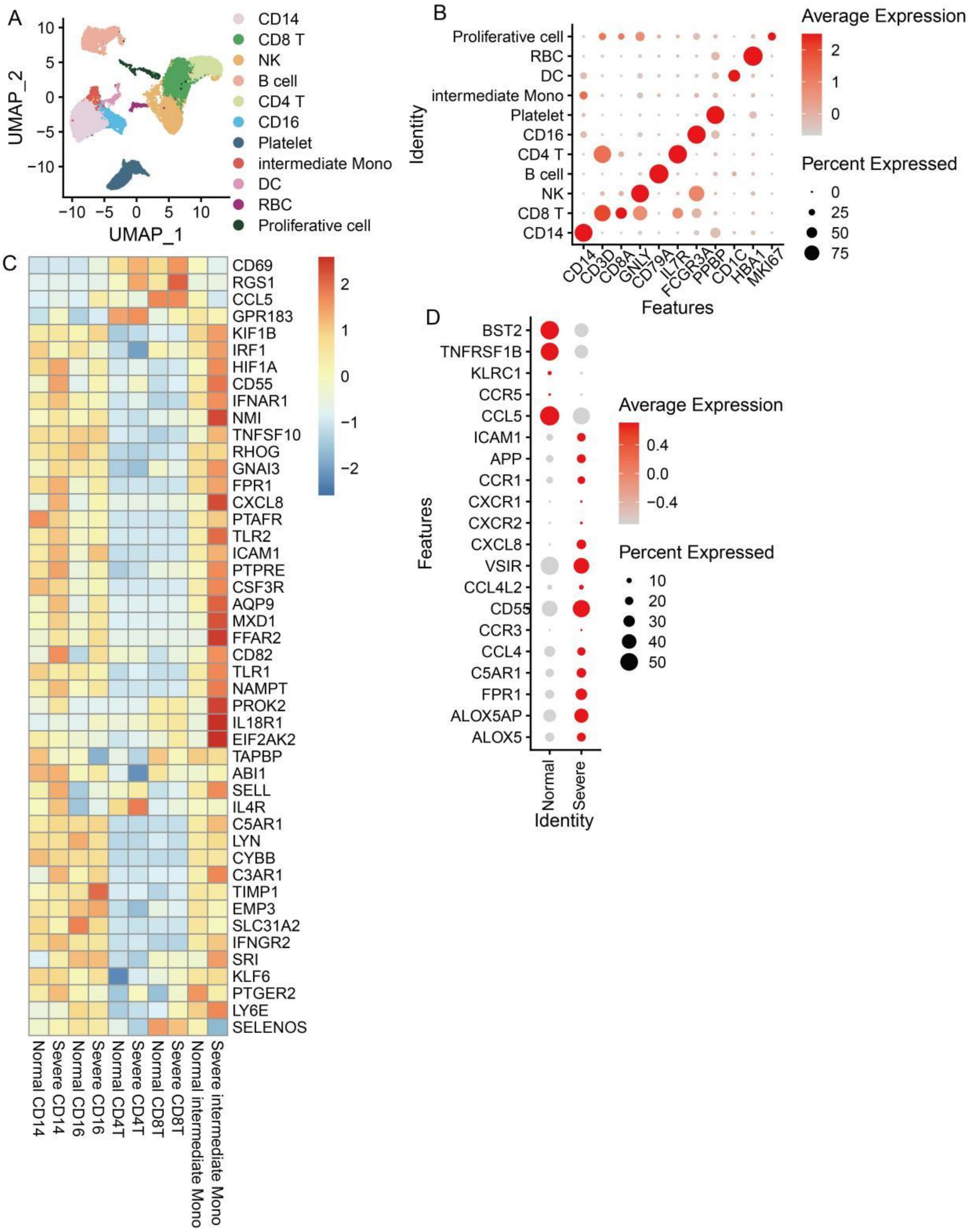

Fig S6. Common PBMC single cell data validation set.

A: UMAP map shows PBMC cell composition of patients with severe COVID-19 and control group. B: The bubble diagram shows the classic markers corresponding to different cell clusters. The figure shows that the identification of cell population has good specificity. C: The expression level of inflammation-related genes in major myeloid cells and lymphocytes of patients with severe COVID-19 and control group. The figure shows the average expression of genes in different cell clusters. D: The expression of significantly changed ligands and receptors was verified in the validation set. The cell type of this data set has been annotated by us. The results of the validation set are basically consistent with the conclusions in the manuscript.

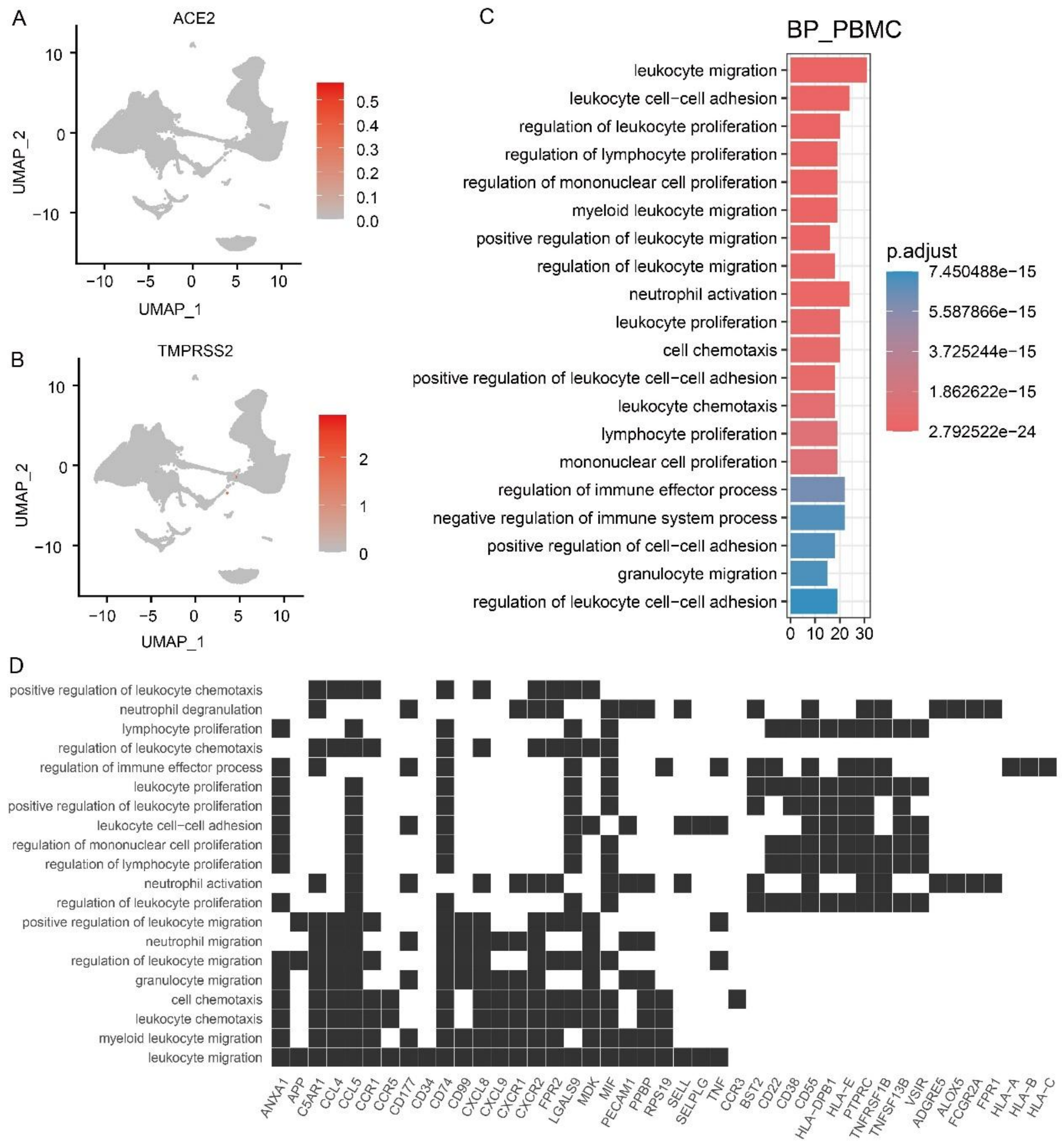

Fig S7. The expression level of ACE2 and TMPRSS2 in blood.

A: The expression level of ACE2 gene in blood. B: The expression level of TMPRSS2 gene in blood. C: The top 20 biological processes enriched by significantly changed receptor and ligand molecules in the blood. D: The enriched heat map shows the significantly changed ligand and receptor genes corresponding to the top 20 biological processes in the blood.

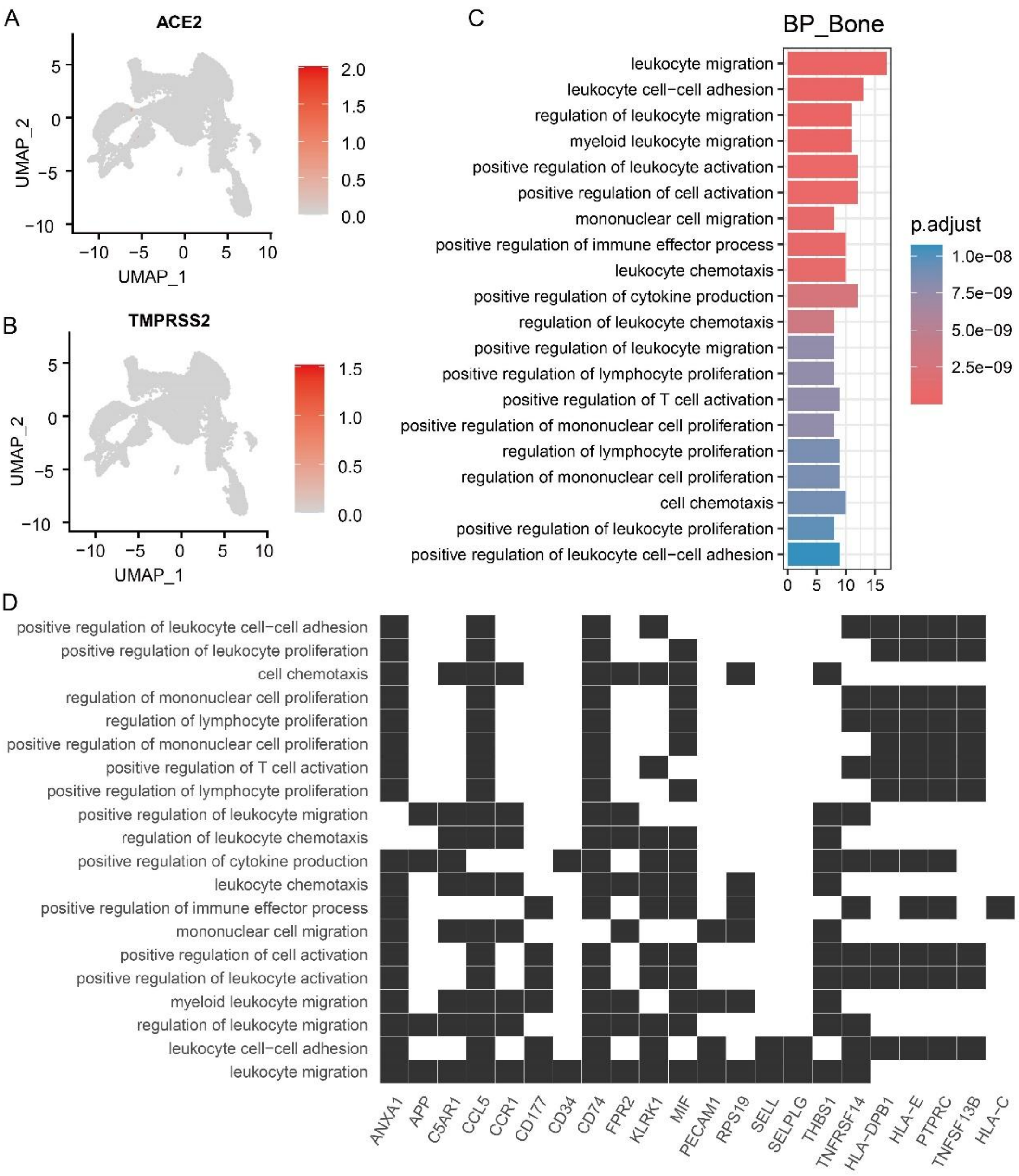

Fig S8. The expression level of ACE2 and TMPRSS2 in bone marrow.

A: The expression level of ACE2 gene in bone marrow. B: The expression level of TMPRSS2 gene in bone marrow. C: The top 20 biological processes enriched by significantly altered receptor and ligand molecules in the bone marrow. D: The enriched heat map shows the significantly changed ligand and receptor genes corresponding to the top 20 biological process in the bone marrow.

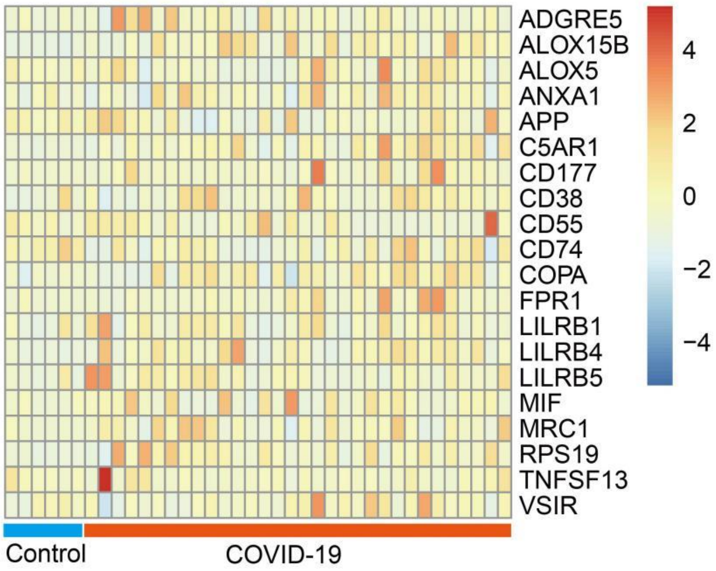

Fig S9. Lung proteomics data from autopsy samples of COVID-19 patients validate the expression of ligand receptors.

Protein expression levels of significantly altered ligands and receptors found in the manuscript were displayed, except for those that were not detected. The protein results showed that these ligands and receptors deduced from the single-cell transcriptome were slightly up-regulated at different levels in the COVID-19 group.

Supplementary table legends:

Supplementary table 1. Clinical diagnostic information of two COVID-19 patients.

Supplementary table 2. Information on antibodies used to detect SARS-CoV-2.

Supplementary table 3. Complete cellular communication ligands and receptors calculated by different tissues.

Supplementary table 4. The age and gender information of single cell data samples used for integration analysis.
